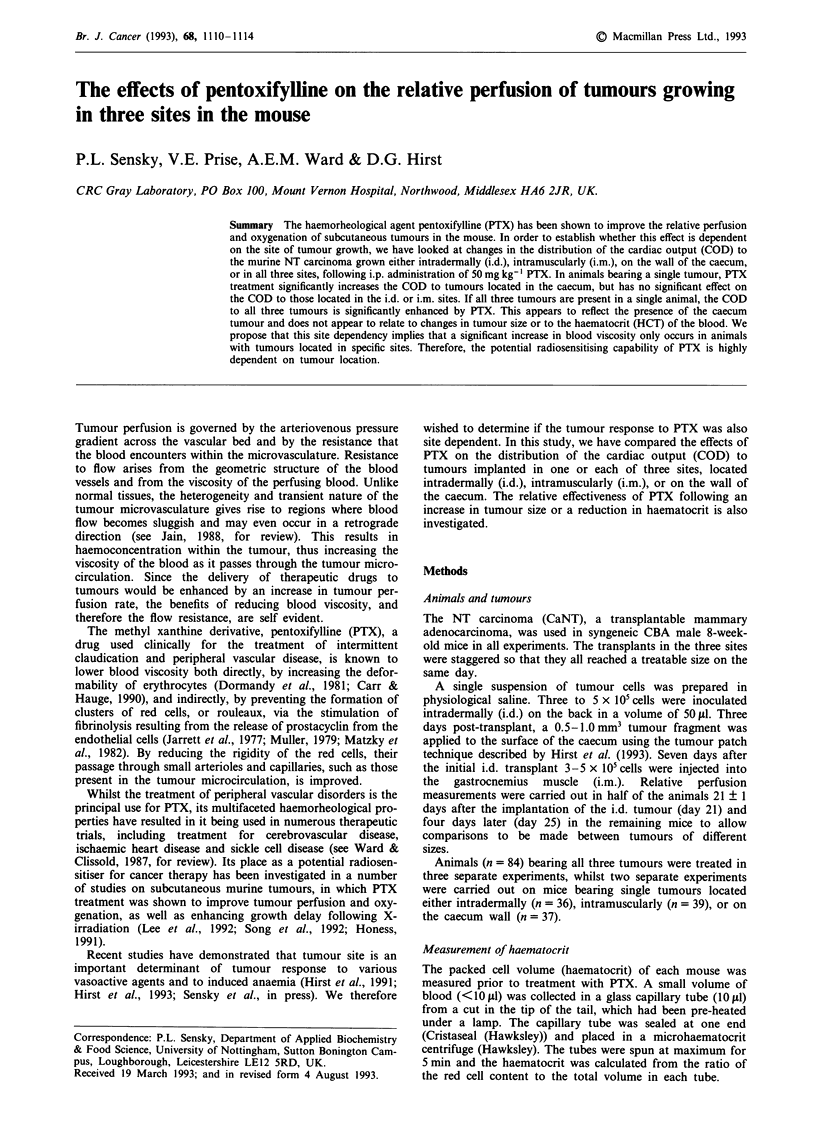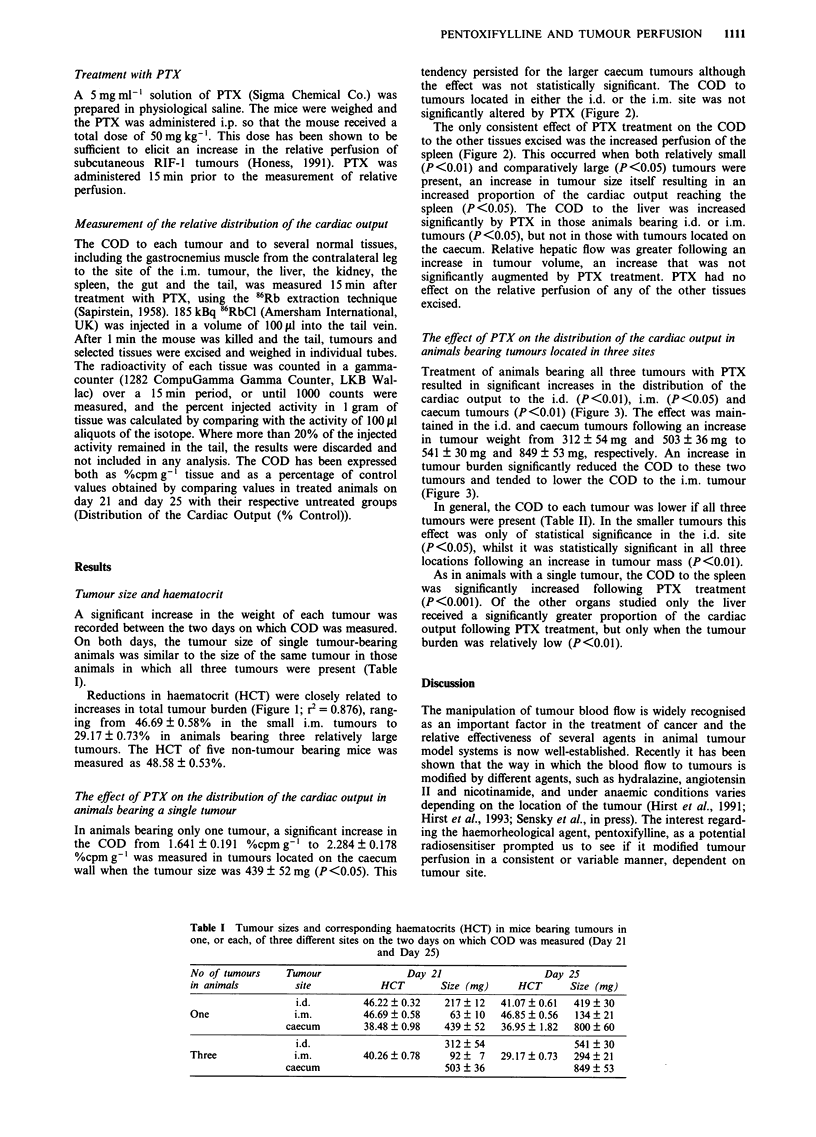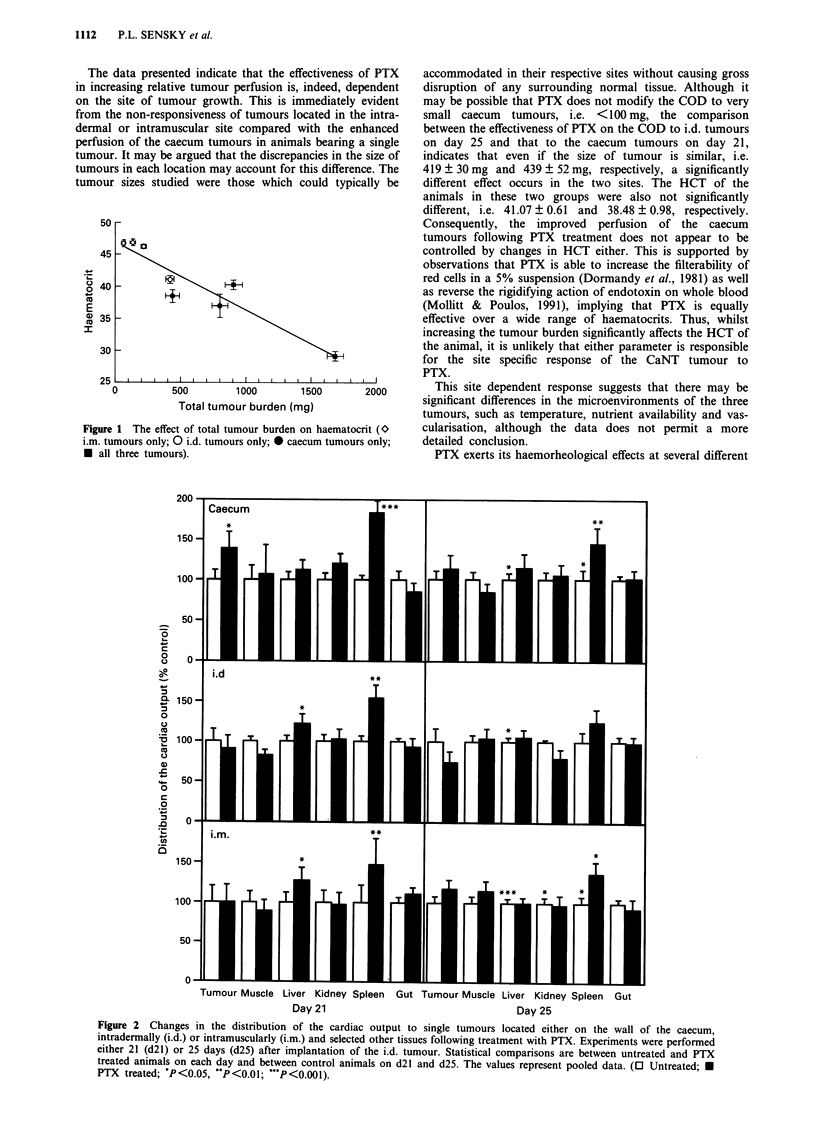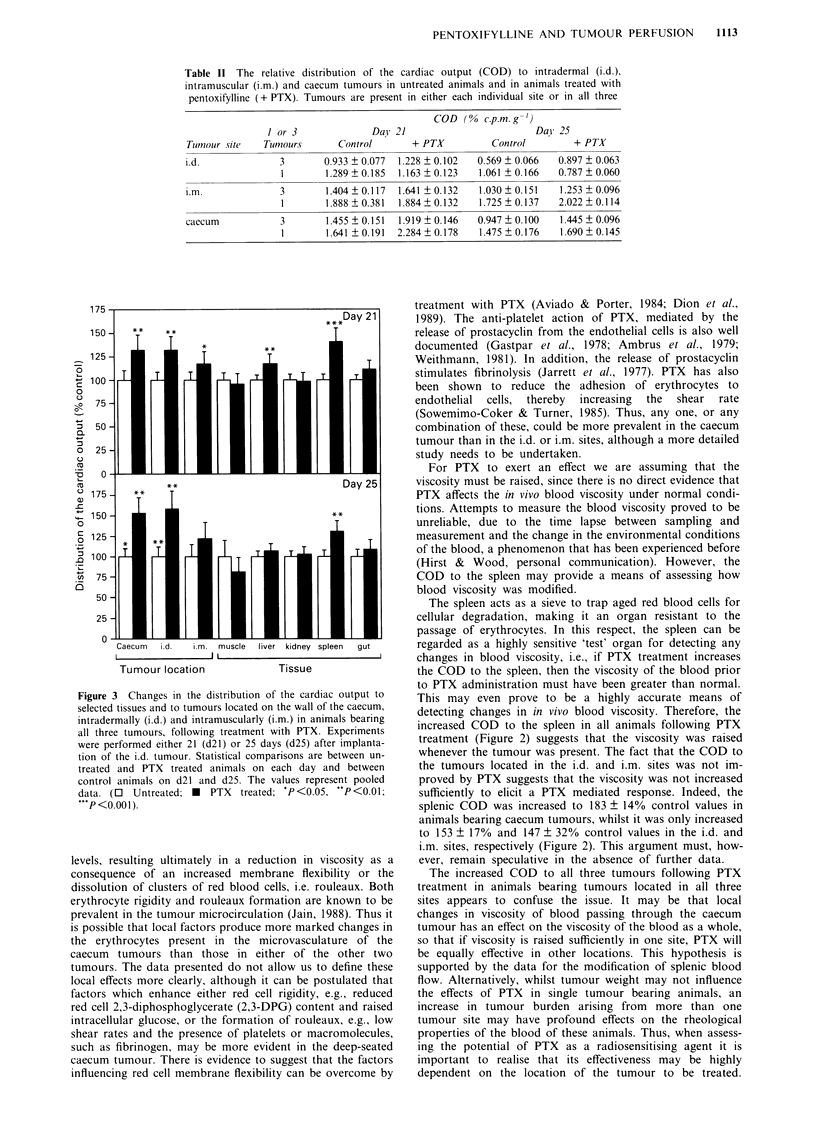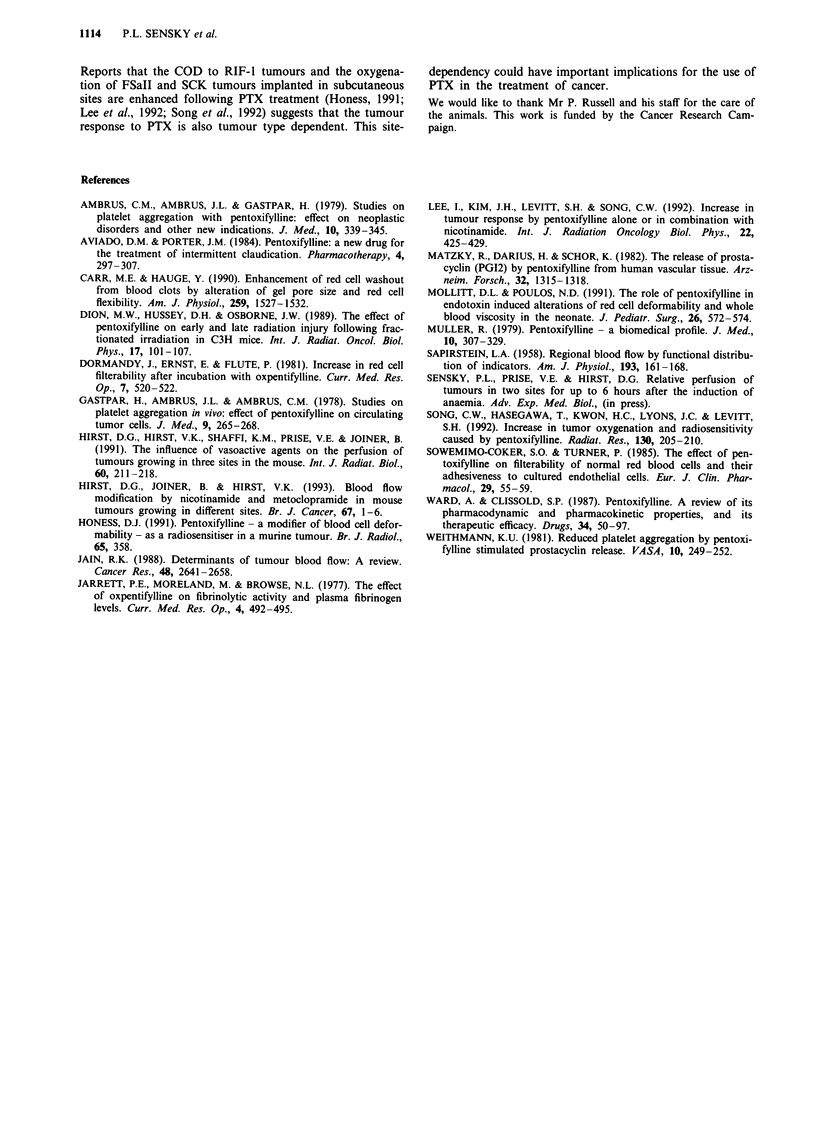# The effects of pentoxifylline on the relative perfusion of tumours growing in three sites in the mouse.

**DOI:** 10.1038/bjc.1993.489

**Published:** 1993-12

**Authors:** P. L. Sensky, V. E. Prise, A. E. Ward, D. G. Hirst

**Affiliations:** CRC Gray Laboratory, Mount Vernon Hospital, Northwood, Middlesex, UK.

## Abstract

The haemorheological agent pentoxifylline (PTX) has been shown to improve the relative perfusion and oxygenation of subcutaneous tumours in the mouse. In order to establish whether this effect is dependent on the site of tumour growth, we have looked at changes in the distribution of the cardiac output (COD) to the murine NT carcinoma grown either intradermally (i.d.), intramuscularly (i.m.), on the wall of the caecum, or in all three sites, following i.p. administration of 50 mg kg-1 PTX. In animals bearing a single tumour, PTX treatment significantly increases the COD to tumours located in the caecum, but has no significant effect on the COD to those located in the i.d. or i.m. sites. If all three tumours are present in a single animal, the COD to all three tumours is significantly enhanced by PTX. This appears to reflect the presence of the caecum tumour and does not appear to relate to changes in tumour size or to the haematocrit (HCT) of the blood. We propose that this site dependency implies that a significant increase in blood viscosity only occurs in animals with tumours located in specific sites. Therefore, the potential radiosensitising capability of PTX is highly dependent on tumour location.


Br. J. Cancer (1993), 68, 1110-1114 © Macmillan Press Ltd., 1993

### The effects of pentoxifylline on the relative perfusion of tumours growing in three sites in the mouse

P.L. Sensky, V.E. Prise, A.E.M. Ward & D.G. Hirst

*CRC Gray Laboratory, PO Box 100, Mount Vernon Hospital, Northwood, Middlesex HA6 2JR, UK.*

**Summary** The haemorheological agent pentoxifylline (PTX) has been shown to improve the relative perfusion and oxygenation of subcutaneous tumours in the mouse. In order to establish whether this effect is dependent on the site of tumour growth, we have looked at changes in the distribution of the cardiac output (COD) to the murine NT carcinoma grown either intradermally (i.d.), intramuscularly (i.m.), on the wall of the caecum, or in all three sites, following i.p. administration of 50 mg $kg^{-1}$ PTX. In animals bearing a single tumour, PTX treatment significantly increases the COD to tumours located in the caecum, but has no significant effect on the COD to those located in the i.d. or i.m. sites. If all three tumours are present in a single animal, the COD to all three tumours is significantly enhanced by PTX. This appears to reflect the presence of the caecum tumour and does not appear to relate to changes in tumour size or to the haematocrit (HCT) of the blood. We propose that this site dependency implies that a significant increase in blood viscosity only occurs in animals with tumours located in specific sites. Therefore, the potential radiosensitising capability of PTX is highly dependent on tumour location.

Tumour perfusion is governed by the arteriovenous pressure gradient across the vascular bed and by the resistance that the blood encounters within the microvasculature. Resistance to flow arises from the geometric structure of the blood vessels and from the viscosity of the perfusing blood. Unlike normal tissues, the heterogeneity and transient nature of the tumour microvasculature gives rise to regions where blood flow becomes sluggish and may even occur in a retrograde direction (see Jain, 1988, for review). This results in haemoconcentration within the tumour, thus increasing the viscosity of the blood as it passes through the tumour microcirculation. Since the delivery of therapeutic drugs to tumours would be enhanced by an increase in tumour perfusion rate, the benefits of reducing blood viscosity, and therefore the flow resistance, are self evident.

The methyl xanthine derivative, pentoxifylline (PTX), a drug used clinically for the treatment of intermittent claudication and peripheral vascular disease, is known to lower blood viscosity both directly, by increasing the deformability of erythrocytes (Dormandy *et al.*, 1981; Carr & Hauge, 1990), and indirectly, by preventing the formation of clusters of red cells, or rouleaux, via the stimulation of fibrinolysis resulting from the release of prostacyclin from the endothelial cells (Jarrett *et al.*, 1977; Muller, 1979; Matzky *et al.*, 1982). By reducing the rigidity of the red cells, their passage through small arterioles and capillaries, such as those present in the tumour microcirculation, is improved.

Whilst the treatment of peripheral vascular disorders is the principal use for PTX, its multifaceted haemorheological properties have resulted in it being used in numerous therapeutic trials, including treatment for cerebrovascular disease, ischaemic heart disease and sickle cell disease (see Ward & Clissold, 1987, for review). Its place as a potential radiosensitiser for cancer therapy has been investigated in a number of studies on subcutaneous murine tumours, in which PTX treatment was shown to improve tumour perfusion and oxygenation, as well as enhancing growth delay following X-irradiation (Lee *et al.*, 1992; Song *et al.*, 1992; Honess, 1991).

Recent studies have demonstrated that tumour site is an important determinant of tumour response to various vasoactive agents and to induced anaemia (Hirst *et al.*, 1991; Hirst *et al.*, 1993; Sensky *et al.*, in press). We therefore wished to determine if the tumour response to PTX was also site dependent. In this study, we have compared the effects of PTX on the distribution of the cardiac output (COD) to tumours implanted in one or each of three sites, located intradermally (i.d.), intramuscularly (i.m.), or on the wall of the caecum. The relative effectiveness of PTX following an increase in tumour size or a reduction in haematocrit is also investigated.

Correspondence: P.L. Sensky, Department of Applied Biochemistry & Food Science, University of Nottingham, Sutton Bonington Campus, Loughborough, Leicestershire LE12 5RD, UK.
Received 19 March 1993; and in revised form 4 August 1993.

### Methods

### *Animals and tumours*

The NT carcinoma (CaNT), a transplantable mammary adenocarcinoma, was used in syngeneic CBA male 8-week-old mice in all experiments. The transplants in the three sites were staggered so that they all reached a treatable size on the same day.

A single suspension of tumour cells was prepared in physiological saline. Three to $5 \times 10^5$ cells were inoculated intradermally (i.d.) on the back in a volume of 50 µl. Three days post-transplant, a 0.5-1.0 $mm^3$ tumour fragment was applied to the surface of the caecum using the tumour patch technique described by Hirst *et al.* (1993). Seven days after the initial i.d. transplant $3-5 \times 10^5$ cells were injected into the gastrocnemius muscle (i.m.). Relative perfusion measurements were carried out in half of the animals 21 ± 1 days after the implantation of the i.d. tumour (day 21) and four days later (day 25) in the remaining mice to allow comparisons to be made between tumours of different sizes.

Animals ($n = 84$) bearing all three tumours were treated in three separate experiments, whilst two separate experiments were carried out on mice bearing single tumours located either intradermally ($n = 36$), intramuscularly ($n = 39$), or on the caecum wall ($n = 37$).

### *Measurement of haematocrit*

The packed cell volume (haematocrit) of each mouse was measured prior to treatment with PTX. A small volume of blood (<10 µl) was collected in a glass capillary tube (10 µl) from a cut in the tip of the tail, which had been pre-heated under a lamp. The capillary tube was sealed at one end (Cristaseal (Hawksley)) and placed in a microhaematocrit centrifuge (Hawksley). The tubes were spun at maximum for 5 min and the haematocrit was calculated from the ratio of the red cell content to the total volume in each tube.

*Treatment with PTX*

A 5 mg ml$^{-1}$ solution of PTX (Sigma Chemical Co.) was prepared in physiological saline. The mice were weighed and the PTX was administered i.p. so that the mouse received a total dose of 50 mg kg$^{-1}$. This dose has been shown to be sufficient to elicit an increase in the relative perfusion of subcutaneous RIF-1 tumours (Honess, 1991). PTX was administered 15 min prior to the measurement of relative perfusion.

*Measurement of the relative distribution of the cardiac output*

The COD to each tumour and to several normal tissues, including the gastrocnemius muscle from the contralateral leg to the site of the i.m. tumour, the liver, the kidney, the spleen, the gut and the tail, was measured 15 min after treatment with PTX, using the $^{86}$Rb extraction technique (Sapirstein, 1958). 185 kBq $^{86}$RbCl (Amersham International, UK) was injected in a volume of 100 µl into the tail vein. After 1 min the mouse was killed and the tail, tumours and selected tissues were excised and weighed in individual tubes. The radioactivity of each tissue was counted in a gamma-counter (1282 CompuGamma Gamma Counter, LKB Wallac) over a 15 min period, or until 1000 counts were measured, and the percent injected activity in 1 gram of tissue was calculated by comparing with the activity of 100 µl aliquots of the isotope. Where more than 20% of the injected activity remained in the tail, the results were discarded and not included in any analysis. The COD has been expressed both as %cpm g$^{-1}$ tissue and as a percentage of control values obtained by comparing values in treated animals on day 21 and day 25 with their respective untreated groups (Distribution of the Cardiac Output (% Control)).

### Results

*Tumour size and haematocrit*

A significant increase in the weight of each tumour was recorded between the two days on which COD was measured. On both days, the tumour size of single tumour-bearing animals was similar to the size of the same tumour in those animals in which all three tumours were present (Table I).

Reductions in haematocrit (HCT) were closely related to increases in total tumour burden (Figure 1; $r^2 = 0.876$), ranging from 46.69 ± 0.58% in the small i.m. tumours to 29.17 ± 0.73% in animals bearing three relatively large tumours. The HCT of five non-tumour bearing mice was measured as 48.58 ± 0.53%.

*The effect of PTX on the distribution of the cardiac output in animals bearing a single tumour*

In animals bearing only one tumour, a significant increase in the COD from 1.641 ± 0.191 %cpm g$^{-1}$ to 2.284 ± 0.178 %cpm g$^{-1}$ was measured in tumours located on the caecum wall when the tumour size was 439 ± 52 mg ($P<0.05$). This tendency persisted for the larger caecum tumours although the effect was not statistically significant. The COD to tumours located in either the i.d. or the i.m. site was not significantly altered by PTX (Figure 2).

The only consistent effect of PTX treatment on the COD to the other tissues excised was the increased perfusion of the spleen (Figure 2). This occurred when both relatively small ($P<0.01$) and comparatively large ($P<0.05$) tumours were present, an increase in tumour size itself resulting in an increased proportion of the cardiac output reaching the spleen ($P<0.05$). The COD to the liver was increased significantly by PTX in those animals bearing i.d. or i.m. tumours ($P<0.05$), but not in those with tumours located on the caecum. Relative hepatic flow was greater following an increase in tumour volume, an increase that was not significantly augmented by PTX treatment. PTX had no effect on the relative perfusion of any of the other tissues excised.

*The effect of PTX on the distribution of the cardiac output in animals bearing tumours located in three sites*

Treatment of animals bearing all three tumours with PTX resulted in significant increases in the distribution of the cardiac output to the i.d. ($P<0.01$), i.m. ($P<0.05$) and caecum tumours ($P<0.01$) (Figure 3). The effect was maintained in the i.d. and caecum tumours following an increase in tumour weight from 312 ± 54 mg and 503 ± 36 mg to 541 ± 30 mg and 849 ± 53 mg, respectively. An increase in tumour burden significantly reduced the COD to these two tumours and tended to lower the COD to the i.m. tumour (Figure 3).

In general, the COD to each tumour was lower if all three tumours were present (Table II). In the smaller tumours this effect was only of statistical significance in the i.d. site ($P<0.05$), whilst it was statistically significant in all three locations following an increase in tumour mass ($P<0.01$).

As in animals with a single tumour, the COD to the spleen was significantly increased following PTX treatment ($P<0.001$). Of the other organs studied only the liver received a significantly greater proportion of the cardiac output following PTX treatment, but only when the tumour burden was relatively low ($P<0.01$).

### Discussion

The manipulation of tumour blood flow is widely recognised as an important factor in the treatment of cancer and the relative effectiveness of several agents in animal tumour model systems is now well-established. Recently it has been shown that the way in which the blood flow to tumours is modified by different agents, such as hydralazine, angiotensin II and nicotinamide, and under anaemic conditions varies depending on the location of the tumour (Hirst *et al.*, 1991; Hirst *et al.*, 1993; Sensky *et al.*, in press). The interest regarding the haemorheological agent, pentoxifylline, as a potential radiosensitiser prompted us to see if it modified tumour perfusion in a consistent or variable manner, dependent on tumour site.

**Table I** Tumour sizes and corresponding haematocrits (HCT) in mice bearing tumours in one, or each, of three different sites on the two days on which COD was measured (Day 21 and Day 25)

| *No of tumours in animals* | *Tumour site* | *Day 21 HCT* | *Day 21 Size (mg)* | *Day 25 HCT* | *Day 25 Size (mg)* |
|---|---|---|---|---|---|
| | i.d. | 46.22 ± 0.32 | 217 ± 12 | 41.07 ± 0.61 | 419 ± 30 |
| One | i.m. | 46.69 ± 0.58 | 63 ± 10 | 46.85 ± 0.56 | 134 ± 21 |
| | caecum | 38.48 ± 0.98 | 439 ± 52 | 36.95 ± 1.82 | 800 ± 60 |
| | i.d. | | 312 ± 54 | | 541 ± 30 |
| Three | i.m. | 40.26 ± 0.78 | 92 ± 7 | 29.17 ± 0.73 | 294 ± 21 |
| | caecum | | 503 ± 36 | | 849 ± 53 |

The data presented indicate that the effectiveness of PTX in increasing relative tumour perfusion is, indeed, dependent on the site of tumour growth. This is immediately evident from the non-responsiveness of tumours located in the intradermal or intramuscular site compared with the enhanced perfusion of the caecum tumours in animals bearing a single tumour. It may be argued that the discrepancies in the size of tumours in each location may account for this difference. The tumour sizes studied were those which could typically be accommodated in their respective sites without causing gross disruption of any surrounding normal tissue. Although it may be possible that PTX does not modify the COD to very small caecum tumours, i.e. <100 mg, the comparison between the effectiveness of PTX on the COD to i.d. tumours on day 25 and that to the caecum tumours on day 21, indicates that even if the size of tumour is similar, i.e. 419 ± 30 mg and 439 ± 52 mg, respectively, a significantly different effect occurs in the two sites. The HCT of the animals in these two groups were also not significantly different, i.e. 41.07 ± 0.61 and 38.48 ± 0.98, respectively. Consequently, the improved perfusion of the caecum tumours following PTX treatment does not appear to be controlled by changes in HCT either. This is supported by observations that PTX is able to increase the filterability of red cells in a 5% suspension (Dormandy et al., 1981) as well as reverse the rigidifying action of endotoxin on whole blood (Mollitt & Poulos, 1991), implying that PTX is equally effective over a wide range of haematocrits. Thus, whilst increasing the tumour burden significantly affects the HCT of the animal, it is unlikely that either parameter is responsible for the site specific response of the CaNT tumour to PTX.

**Figure 1** The effect of total tumour burden on haematocrit (◇ i.m. tumours only; ○ i.d. tumours only; ● caecum tumours only; ■ all three tumours).

This site dependent response suggests that there may be significant differences in the microenvironments of the three tumours, such as temperature, nutrient availability and vascularisation, although the data does not permit a more detailed conclusion.

PTX exerts its haemorheological effects at several different

**Figure 2** Changes in the distribution of the cardiac output to single tumours located either on the wall of the caecum, intradermally (i.d.) or intramuscularly (i.m.) and selected other tissues following treatment with PTX. Experiments were performed either 21 (d21) or 25 days (d25) after implantation of the i.d. tumour. Statistical comparisons are between untreated and PTX treated animals on each day and between control animals on d21 and d25. The values represent pooled data. (□ Untreated; ■ PTX treated; *$P<0.05$, **$P<0.01$; ***$P<0.001$).

**Table II** The relative distribution of the cardiac output (COD) to intradermal (i.d.), intramuscular (i.m.) and caecum tumours in untreated animals and in animals treated with pentoxifylline (+ PTX). Tumours are present in either each individual site or in all three

| | | COD (% c.p.m. g$^{-1}$) | | | |
|---|---|---|---|---|---|
| | *1 or 3* | *Day 21* | | *Day 25* | |
| *Tumour site* | *Tumours* | *Control* | *+ PTX* | *Control* | *+ PTX* |
| i.d. | 3 | 0.933 ± 0.077 | 1.228 ± 0.102 | 0.569 ± 0.066 | 0.897 ± 0.063 |
| | 1 | 1.289 ± 0.185 | 1.163 ± 0.123 | 1.061 ± 0.166 | 0.787 ± 0.060 |
| i.m. | 3 | 1.404 ± 0.117 | 1.641 ± 0.132 | 1.030 ± 0.151 | 1.253 ± 0.096 |
| | 1 | 1.888 ± 0.381 | 1.884 ± 0.132 | 1.725 ± 0.137 | 2.022 ± 0.114 |
| caecum | 3 | 1.455 ± 0.151 | 1.919 ± 0.146 | 0.947 ± 0.100 | 1.445 ± 0.096 |
| | 1 | 1.641 ± 0.191 | 2.284 ± 0.178 | 1.475 ± 0.176 | 1.690 ± 0.145 |

**Figure 3** Changes in the distribution of the cardiac output to selected tissues and to tumours located on the wall of the caecum, intradermally (i.d.) and intramuscularly (i.m.) in animals bearing all three tumours, following treatment with PTX. Experiments were performed either 21 (d21) or 25 days (d25) after implantation of the i.d. tumour. Statistical comparisons are between untreated and PTX treated animals on each day and between control animals on d21 and d25. The values represent pooled data. (□ Untreated; ■ PTX treated; *$P<0.05$, **$P<0.01$; ***$P<0.001$).

levels, resulting ultimately in a reduction in viscosity as a consequence of an increased membrane flexibility or the dissolution of clusters of red blood cells, i.e. rouleaux. Both erythrocyte rigidity and rouleaux formation are known to be prevalent in the tumour microcirculation (Jain, 1988). Thus it is possible that local factors produce more marked changes in the erythrocytes present in the microvasculature of the caecum tumours than those in either of the other two tumours. The data presented do not allow us to define these local effects more clearly, although it can be postulated that factors which enhance either red cell rigidity, e.g., reduced red cell 2,3-diphosphoglycerate (2,3-DPG) content and raised intracellular glucose, or the formation of rouleaux, e.g., low shear rates and the presence of platelets or macromolecules, such as fibrinogen, may be more evident in the deep-seated caecum tumour. There is evidence to suggest that the factors influencing red cell membrane flexibility can be overcome by treatment with PTX (Aviado & Porter, 1984; Dion *et al.*, 1989). The anti-platelet action of PTX, mediated by the release of prostacyclin from the endothelial cells is also well documented (Gastpar *et al.*, 1978; Ambrus *et al.*, 1979; Weithmann, 1981). In addition, the release of prostacyclin stimulates fibrinolysis (Jarrett *et al.*, 1977). PTX has also been shown to reduce the adhesion of erythrocytes to endothelial cells, thereby increasing the shear rate (Sowemimo-Coker & Turner, 1985). Thus, any one, or any combination of these, could be more prevalent in the caecum tumour than in the i.d. or i.m. sites, although a more detailed study needs to be undertaken.

For PTX to exert an effect we are assuming that the viscosity must be raised, since there is no direct evidence that PTX affects the *in vivo* blood viscosity under normal conditions. Attempts to measure the blood viscosity proved to be unreliable, due to the time lapse between sampling and measurement and the change in the environmental conditions of the blood, a phenomenon that has been experienced before (Hirst & Wood, personal communication). However, the COD to the spleen may provide a means of assessing how blood viscosity was modified.

The spleen acts as a sieve to trap aged red blood cells for cellular degradation, making it an organ resistant to the passage of erythrocytes. In this respect, the spleen can be regarded as a highly sensitive 'test' organ for detecting any changes in blood viscosity, i.e., if PTX treatment increases the COD to the spleen, then the viscosity of the blood prior to PTX administration must have been greater than normal. This may even prove to be a highly accurate means of detecting changes in *in vivo* blood viscosity. Therefore, the increased COD to the spleen in all animals following PTX treatment (Figure 2) suggests that the viscosity was raised whenever the tumour was present. The fact that the COD to the tumours located in the i.d. and i.m. sites was not improved by PTX suggests that the viscosity was not increased sufficiently to elicit a PTX mediated response. Indeed, the splenic COD was increased to 183 ± 14% control values in animals bearing caecum tumours, whilst it was only increased to 153 ± 17% and 147 ± 32% control values in the i.d. and i.m. sites, respectively (Figure 2). This argument must, however, remain speculative in the absence of further data.

The increased COD to all three tumours following PTX treatment in animals bearing tumours located in all three sites appears to confuse the issue. It may be that local changes in viscosity of blood passing through the caecum tumour has an effect on the viscosity of the blood as a whole, so that if viscosity is raised sufficiently in one site, PTX will be equally effective in other locations. This hypothesis is supported by the data for the modification of splenic blood flow. Alternatively, whilst tumour weight may not influence the effects of PTX in single tumour bearing animals, an increase in tumour burden arising from more than one tumour site may have profound effects on the rheological properties of the blood of these animals. Thus, when assessing the potential of PTX as a radiosensitising agent it is important to realise that its effectiveness may be highly dependent on the location of the tumour to be treated.

Reports that the COD to RIF-1 tumours and the oxygenation of FSaII and SCK tumours implanted in subcutaneous sites are enhanced following PTX treatment (Honess, 1991; Lee *et al.*, 1992; Song *et al.*, 1992) suggests that the tumour response to PTX is also tumour type dependent. This site-dependency could have important implications for the use of PTX in the treatment of cancer.

We would like to thank Mr P. Russell and his staff for the care of the animals. This work is funded by the Cancer Research Campaign.

### References

AMBRUS, C.M., AMBRUS, J.L. & GASTPAR, H. (1979). Studies on platelet aggregation with pentoxifylline: effect on neoplastic disorders and other new indications. *J. Med.*, **10**, 339-345.

AVIADO, D.M. & PORTER, J.M. (1984). Pentoxifylline: a new drug for the treatment of intermittent claudication. *Pharmacotherapy*, **4**, 297-307.

CARR, M.E. & HAUGE, Y. (1990). Enhancement of red cell washout from blood clots by alteration of gel pore size and red cell flexibility. *Am. J. Physiol.*, **259**, 1527-1532.

DION, M.W., HUSSEY, D.H. & OSBORNE, J.W. (1989). The effect of pentoxifylline on early and late radiation injury following fractionated irradiation in C3H mice. *Int. J. Radiat. Oncol. Biol. Phys.*, **17**, 101-107.

DORMANDY, J., ERNST, E. & FLUTE, P. (1981). Increase in red cell filterability after incubation with oxpentifylline. *Curr. Med. Res. Op.*, **7**, 520-522.

GASTPAR, H., AMBRUS, J.L. & AMBRUS, C.M. (1978). Studies on platelet aggregation *in vivo*: effect of pentoxifylline on circulating tumor cells. *J. Med.*, **9**, 265-268.

HIRST, D.G., HIRST, V.K., SHAFFI, K.M., PRISE, V.E. & JOINER, B. (1991). The influence of vasoactive agents on the perfusion of tumours growing in three sites in the mouse. *Int. J. Radiat. Biol.*, **60**, 211-218.

HIRST, D.G., JOINER, B. & HIRST, V.K. (1993). Blood flow modification by nicotinamide and metoclopramide in mouse tumours growing in different sites. *Br. J. Cancer*, **67**, 1-6.

HONESS, D.J. (1991). Pentoxifylline - a modifier of blood cell deformability - as a radiosensitiser in a murine tumour. *Br. J. Radiol.*, **65**, 358.

JAIN, R.K. (1988). Determinants of tumour blood flow: A review. *Cancer Res.*, **48**, 2641-2658.

JARRETT, P.E., MORELAND, M. & BROWSE, N.L. (1977). The effect of oxpentifylline on fibrinolytic activity and plasma fibrinogen levels. *Curr. Med. Res. Op.*, **4**, 492-495.

LEE, I., KIM, J.H., LEVITT, S.H. & SONG, C.W. (1992). Increase in tumour response by pentoxifylline alone or in combination with nicotinamide. *Int. J. Radiation Oncology Biol. Phys.*, **22**, 425-429.

MATZKY, R., DARIUS, H. & SCHOR, K. (1982). The release of prostacyclin (PGI2) by pentoxifylline from human vascular tissue. *Arzneim. Forsch.*, **32**, 1315-1318.

MOLLITT, D.L. & POULOS, N.D. (1991). The role of pentoxifylline in endotoxin induced alterations of red cell deformability and whole blood viscosity in the neonate. *J. Pediatr. Surg.*, **26**, 572-574.

MULLER, R. (1979). Pentoxifylline - a biomedical profile. *J. Med.*, **10**, 307-329.

SAPIRSTEIN, L.A. (1958). Regional blood flow by functional distribution of indicators. *Am. J. Physiol.*, **193**, 161-168.

SENSKY, P.L., PRISE, V.E. & HIRST, D.G. Relative perfusion of tumours in two sites for up to 6 hours after the induction of anaemia. *Adv. Exp. Med. Biol.*, (in press).

SONG, C.W., HASEGAWA, T., KWON, H.C., LYONS, J.C. & LEVITT, S.H. (1992). Increase in tumor oxygenation and radiosensitivity caused by pentoxifylline. *Radiat. Res.*, **130**, 205-210.

SOWEMIMO-COKER, S.O. & TURNER, P. (1985). The effect of pentoxifylline on filterability of normal red blood cells and their adhesiveness to cultured endothelial cells. *Eur. J. Clin. Pharmacol.*, **29**, 55-59.

WARD, A. & CLISSOLD, S.P. (1987). Pentoxifylline. A review of its pharmacodynamic and pharmacokinetic properties, and its therapeutic efficacy. *Drugs*, **34**, 50-97.

WEITHMANN, K.U. (1981). Reduced platelet aggregation by pentoxifylline stimulated prostacyclin release. *VASA*, **10**, 249-252.